# Non-Invasive Radiofrequency-Induced Targeted Hyperthermia for the Treatment of Hepatocellular Carcinoma

**DOI:** 10.4061/2011/676957

**Published:** 2011-05-29

**Authors:** Mustafa Raoof, Steven A. Curley

**Affiliations:** ^1^Department of Surgical Oncology, Rice University, Houston, TX 77030, USA; ^2^Department of Mechanical Engineering Materials Science, Rice University, 1400 Holcombe Boulevard, Unit 444, Houston, TX 77030, USA

## Abstract

Targeted biological therapies for hepatocellular cancer have shown minimal improvements in median survival. Multiple pathways to oncogenesis leading to rapid development of resistance to such therapies is a concern. Non-invasive radiofrequency field-induced targeted hyperthermia using nanoparticles is a radical departure from conventional modalities. In this paper we underscore the need for innovative strategies for the treatment of hepatocellular cancer, describe the central paradigm of targeted hyperthermia using non-invasive electromagnetic energy, review the process of characterization and modification of nanoparticles for the task, and summarize data from cell-based and animal-based models of hepatocellular cancer treated with non-invasive RF energy. Finally, future strategies and challenges in bringing this modality from bench to clinic are discussed.

## 1. Introduction

Hepatocellular cancer (HCC) presents a global challenge. It is the sixth most common cancer and the third most common cause of cancer-related deaths worldwide [1]. The incidence of HCC is on the rise. It is estimated that approximately 1 million new cases of HCC are diagnosed each year [2]. Chronic infection with hepatitis B and hepatitis C virus coupled with other risk factors such as diabetes, obesity, smoking, and heavy alcohol consumption contribute to this rising incidence [3]. Growing burden of disease presents a significant problem, as majority of patients diagnosed with HCC cannot be treated with curative intent [4]. This is because of delay in diagnosis and concomitant hepatic dysfunction. Worldwide, the median survival of patients with advanced HCC who remain untreated is less than 4 months [5]. 

Surgical resection and transplantation yield 5-year survivals ranging from 35% to over 70% [4, 6–10]. These therapies are suited for few candidates that have limited local disease and fit into a strict clinical criteria. For other patients with HCC, treatment options include intratumoral injection of absolute ethanol or acetic acid, invasive thermal destruction using microwave or radiofrequency needles and transarterial chemoembolization (TACE) using drug-eluting beads. Considered together, local-regional therapies have lead to a modest increase in median survival [11].

While targeted biological therapies such as monoclonal antibodies have been successful in treating other cancers, HCC remains a challenge. Recently Sorafenib, a multikinase inhibitor, has shown an improvement in median survival of 2.3–2.8 months compared to placebo in clinical trials [12, 13]. Futility of biological therapies is because of multiple pathways to oncogenesis in HCC and rapid development of resistance to these agents. Non-invasive electromagnetic field-induced targeted hyperthermia for the treatment of HCC is a radical departure from traditional therapies and holds immense potential.

Electromagnetic energy in the form of near-infrared (NIR) photothermal energy, inductively coupled magnetic field or radiofrequency field, has been employed to deliver non-invasive targeted hyperthermia to malignant cells [14–17]. The rationale for such therapies is based on the observation that metal nanoparticles targeted to tumor cells generate heat when exposed to electromagnetic energy causing them to undergo heat-stress-triggered apoptosis while sparing normal tissues. The use of non-invasive NIR energy to produce photothermal toxicity is limited by its low tissue penetrance and hence inability to treat deeper lesions as in HCC [18]. Use of inductively coupled magnetic fields to heat charged magnetic dextran-coated metal nanoparticles such as iron oxide (Fe_3_O_4_) has also been demonstrated. However, thermal enhancement is limited by the magnetic field strength applicable to abdominal tumors (<4.5 kA/m, 100 kHz) and by difficulty in targeting magnetic nanoparticles to malignant cells [16, 17]. In contrast, non-invasive radiofrequency field-induced heating of metal nanoparticles offers several advantages over others in the treatment of HCC, as detailed later. The purpose of this paper is to summarize current strategies for delivering non-invasive radiofrequency field-mediated hyperthermia to malignant cells and its application to HCC.

## 2. Radio Waves in the Treatment of Cancer

Radio waves are low-frequency electromagnetic waves that have low tissue-specific absorption rate (SAR) and, therefore, excellent whole body tissue penetration. Radio waves are considered safe with several studies reporting no harmful effects in humans exposed to RF field for several hours [19, 20]. Because of their excellent safety profile, radio waves have been widely utilized in medicine including communication devices, diagnostic imaging, and ablation therapies. 

**Figure 1:**
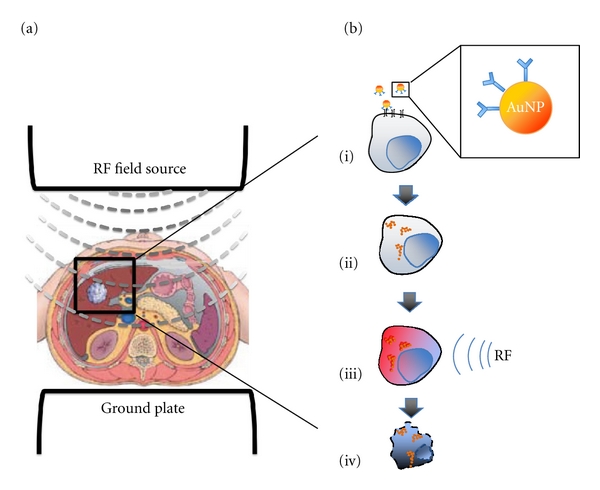
Principle of the non-invasive RF based treatment of HCC. A. RF field source is used to generate a uniform low frequency electromagnetic field that penetrates tissues and reaches the tumor. B. Nanoparticles that can be thermally activated are conjugated to monoclonal antibodies against known targets expressed on HCC (i), internalized specifically by cancer cells after systemic administration (ii), and upon RF activation release heat (iii) causing targeted cancer cell death.

Radiofrequency ablation (RFA) has particularly been effective for local regional control of HCC in patients not amenable to surgical resection or awaiting transplantation. This technique requires high RF energy transfer from an electrode placed within the tumor percutaneously or intraoperatively under image guidance. Energy dissipated through the RF electrode causes coagulative necrosis and thermal destruction of the tumor [21]. In contrast to RFA, nanoparticle targeted hyperthermia is a non-invasive approach to deliver hyperthermia at a cellular level without harming surrounding normal tissue (Figure 1).

## 3. Kanzius RF Generator

**Figure 2:**
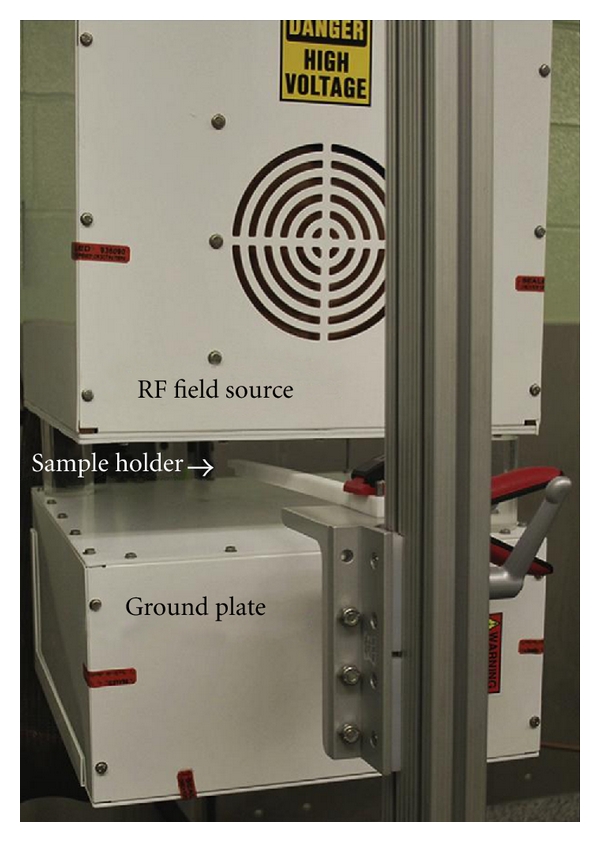
Kanzius RF generator: RF field source transmission antenna (Tx head) and ground plate (Reciprocal Rx head) are separated by 10 cm air-gap. Samples are placed 2.5 cm from the Tx head on the Teflon holder.

Non-invasive radiofrequency-based hyperthermia, unlike radiofrequency ablation, requires an external radiofrequency field generator (Kanzius RF generator) [14, 22, 23]. This is a variable power (0–2 KW) 13.56 MHz RF field generator (Therm Med LLC, Erie, Pennsylvania). The RF generator is connected to a high Q coupling system with a Tx head (focused end-fired antenna circuit) and reciprocal Rx head (as a return for the generator) mounted on a swivel bracket allowing the RF field to be oriented in either a horizontal or vertical direction (Figure 2). The distance between the two heads is adjustable. The coaxial end-fire circuit in the Tx head produces an electronic focused RF field up to 15 cm in diameter. The electromagnetic field strength between the Tx and Rx head is established and calibrated in a Faraday-shielded room to exclude any interference from external RF sources. The field is measured using a Hewlett Packard Spectrum Analyzer (model 8566B, Agilent, Santa Clara, CA), an isotropic field monitor, and a probe (models FM2004 and FP2000, Amplifier Research Inc., Souderton, PA). Utilizing an output power of 600 W, maximum electric field strength (Ep) of 12.4 kV/m is measured at a distance 2.5 cm from the Tx head.

## 4. Radiofrequency-Induced Heating of Nanoparticles

We have demonstrated heating of several nanoparticles in the RF field including gold nanoparticles (AuNP), gold silica nanoshells, single-walled carbon nanotubes (SWNT), and water-soluble derivatives of C60 fullerenes [14, 22–27]. A treatment strategy based on molecular targeting of gold and carbon nanoparticles has several advantages: they are simple and inexpensive to synthesize; they are easily characterized due to their signature optical absorptions; their surface chemistry readily permits manipulation of charge and shape and attaching cancer cell-targeting molecules, including antibodies, peptides, or pharmacologic agents, is easily achieved. A detailed discussion on the methods utilized to characterize and synthesize such nanoparticles is beyond the scope of this paper and is described elsewhere.

**Figure 3:**
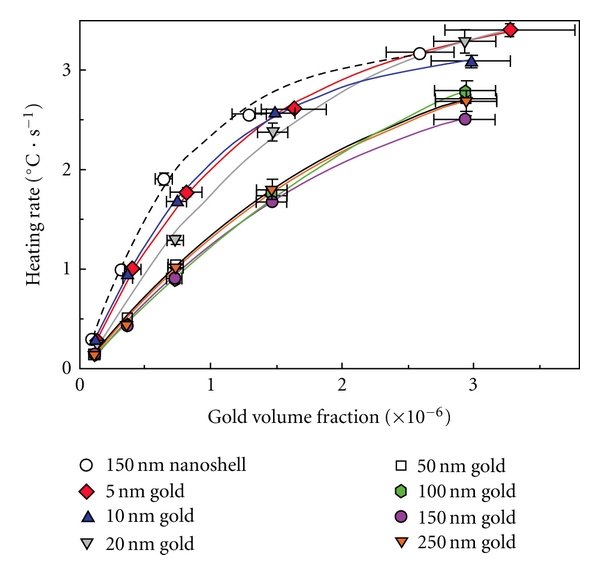
Size-dependent heating of gold nanoparticles in non-invasive RF field. 150 nm gold nanoshells (a shell thickness of 10–15 nm) demonstrate heating rates similar to 5 nm gold nanoparticles. *Reproduced with permission from Nano Research, vol. 2, no. 5, pp. 400-405, 2009. *

Heating of AuNPs in RF field is concentration and size dependent as shown in Figure 3. AuNPs with small diameters (5 nm) heat better than larger particles. In our previous papers we have explained the increased heating of smaller particles on the basis of increased ohmic dissipation with increased resistivity of smaller particles [28]. The exact physical basis of heat generation by nanoparticles is not entirely clear and is an area of active investigation.

**Figure 4:**
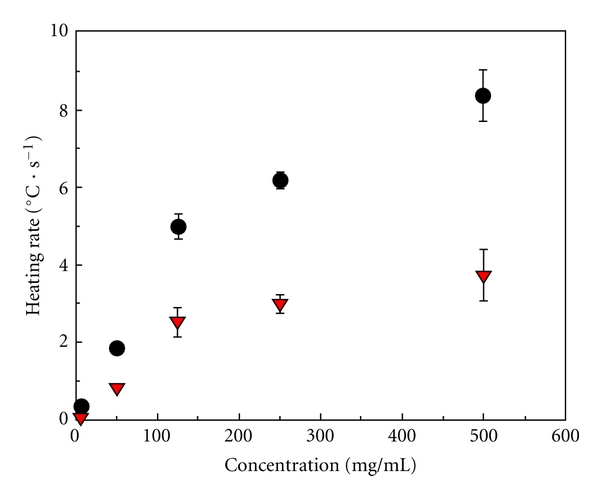
Concentration-dependent heating rate of Kentera SWNT suspensions under 600 W of RF generator output power. Suspensions exhibited nonlinear heating rates with increasing SWNT concentration. Shown are the averages of the heating rates for Kentera SWNTs (dots) and the calculated SWNT heating contribution (triangles). *Reproduced with permission from Cancer, vol. 110, no. 12, pp. 2654–2665, 2007. *

Similar to AuNPs, single-walled carbon nanotubes (SWNT) functionalized with a biocompatible nonionic polymer (Kentera) demonstrate a linear rise in temperature after RF activation [26]. The heating rate also increases linearly with RF generator output power. However, the heating rate of SWNT suspensions increases nonlinearly with increasing concentrations (Figure 4).

## 5. Targeting Strategies

In order to deliver targeted hyperthermia to cancer cells it is crucial that the nanoparticles are modified to specifically enhance uptake by tumor cells. In our studies, we have conjugated monoclonal antibodies (raised against tumor-specific targets) to nanoparticles. Two approaches are described here using chimeric anti-epidermal growth factor receptor (EGFR) antibody (or C225) conjugation to AuNPs as a prototypical example.

**Noncovalent Conjugation**

Colloidal suspensions of spherical AuNPs are stabilized during synthesis using citrate as a stabilizing agent to prevent aggregation. For the purposes of conjugation the AuNPs are concentrated to remove citrate. These are then resuspended in a buffer solution whose pH matches the isoelectric point of the monoclonal antibody (pH = 8.5 for C225). The monoclonal antibody is then slowly added to AuNP colloidal suspension in a (w/w) ratio of 20 : 50 and gently mixed. Surface modification is confirmed by <10 nm red shift in UV-Vis peak absorption spectra of modified AuNPs. While easy to perform, NonCovalent conjugation is nondirectional. Moreover, other proteins in biological samples can replace surface-bound antibodies.

**Thiol-Based Covalent Linkage**

Near covalent bonds can be formed on the surface of AuNP when monoclonal antibodies are attached to linker with a thiol functional group. Thiol-based conjugation is stronger and more stable than NonCovalent electrostatic interaction and hence preferred. Directional conjugation also presents Fab portion of the antibody to the tumor antigens maximizing receptor-ligand interaction. We have employed methods described by Kumar et al. with slight modifications [29].

## 6. *In Vitro* Thermal Cytotoxicity of RF-Induced Hyperthermia

We have demonstrated the effect of RF treatment on various heptaocellular cancer cell lines using different nanoparticles. Hep3B and HepG2 cells treated with kentera modified SWNT were exposed to an 800 W RF field [26]. Significant thermal cytotoxicity was demonstrated with 2 minutes of RF exposure in a concentration-dependent manner. Higher concentrations (500 mg/L) produced 100% thermal cytotoxicity in comparison to untreated controls (11%) and cells treated with kentera solution only (35%), *P* value <.01.

In separate experiments, Hep3B cells were exposed to naked 5 nm AuNPs at 1, 10, or 67 *μ*M for 24 hours [27]. The gold containingmedium was aspirated and replaced by fresh medium. Cells were then exposed to RF treatment for 1, 2, and 5 minutes. The resulting thermal cytotoxicity is summarized in Table 1. This RF-induced gold nanoparticle-based thermal cytotoxicity was concentration dependent (data not shown). 

These experiments indicate that non-invasive RF field-based hyperthermia using untargeted nanoparticles is highly effective in treating HCC cell lines. In separate experiments, a more targeted approach was implemented. Panc-1 cells treated with C225-conjugated AuNP and exposed to RF field in RF showed higher thermal cytotoxicity than cells treated with naked gold nanoparticles and exposed to RF field (data in press). Using a similar approach for HCC presents challenges. Unlike pancreatic cancer, expression of EGFR on HCC cell lines is moderate at best. A systematic investigation to identify better and more specific molecular targets on HCC is currently underway.

## 7. Tumor Response to RF-Induced Hyperthermia in Animal Models

In order to demonstrate that SWNT localized to VX2 tumor can be remotely activated by RF field to produce thermal cytotoxicity, adult New Zealand white rabbits bearing orthotopic VX2 tumors ranging in size from 1.0 cm to 1.3 cm in greatest dimension underwent a direct intratumoral injection of water-soluble SWNTs or control solutions [26]. Rabbits were treated with or without RF for 2 minutes. Two days after RF treatment, all animals were sacrificed. Histopathology sections from tumors injected with SWNTs revealed complete thermal necrosis of the tumor tissue with a surrounding 2 mm to 5 mm zone of thermal injury to the liver. There was no evidence of nonspecific injury to other organs and tissues. Tumors that had been injected with SWNTs but not treated with RF also were completely viable, as were tumors that had not been injected with SWNTs or control solutions and had been treated with RF alone (data not shown).

As a next step and to develop an entirely non-invasive treatment modality, our goal was to inject antibody-conjugated nanoparticles systemically and allow them to concentrate specifically in the tumor tissue. To investigate that further and as a proof of principle, we used an ectopic murine model of EGFR expressing Panc-1 tumors. This is because of ease of availability and extensive characterization of chimeric C225. C225 was directionally conjugated to 10nm AuNPs. The conjugates when systemically injected concentrated specifically to the tumor site unlike nontargeted AuNPs. Weekly cycles of injection of nanoconjugates followed by RF treatment for 10 minutes halted the growth of tumors during 7 weeks of treatment compared to RF only, nanoconjugates only, and untreated controls (*P* < .004), and in some cases produced a complete response (data in press). In these *in vivo *experiments, no untoward or unexplained side effects were noted. We expect that as HCC-specific tumor targets are identified, a similar approach can be employed.

## 8. Future Direction and Challenges

In this brief paper, we have summarized the grand opportunities and challenges, non-invasive RF-based treatment of HCC presents. There are several advantages of using such an approach. Radio waves have well-documented safety in humans, mainly because of their minimal SAR. At the same time they have excellent whole body penetration reaching tumors in every possible location. Nanoparticles used to harness RF electromagnetic energy within the tumor are inexpensive and simple to synthesize with high reproducibility. They can be easily characterized using well-established methods. They are sufficiently small to navigate through the most compromised tumor vasculature. In addition to this, AuNPs are biocompatible, have not been associated with any acute or chronic toxicity in preclinical studies, and are already used clinically to treat severe rheumatoid arthritis. These features make it an attractive, safe, and effective treatment modality for HCC patients including those with hepatic dysfunction.

Identification of HCC-specific tumor targets is an area of active research inquiry. Attempts to characterize immunologic differences between human HCC cells and normal hepagtocytes led to the development of AF-20 and FB-50 monoclonal antibodies that recognize different domains on overexpressed aspartyl *β*-hydroxylase. However, these monoclonal antibodies are not commercially available, and their receptor-ligand interaction remains to be characterized. Similarly, another challenge is *in vivo* thermal dosimetry. The need to measure real-time temperature for treatments that employ hyperthermia has led to the development of magnetic resonance thermography. However it seems plausible that the interaction of the two magnetic fields will not allow utilization of magnetic resonance thermography for measurement of RF-based hyperthermia. Other techniques that allow real-time thermography need to be developed. Finally, we anticipate that long-term RF-induced hyperthermia-based treatment for HCC has the potential to induce thermotolerance as well as thermoresistance in some subsets of patients. Such patients may benefit from enhancing effects of chemotherapeutic agents using RF-based hyperthermia, which should also be investigated in the future.

##  Funding

This work was funded from the NIH (U54CA143837), NIH M. D. Anderson Cancer Center Support Grant CA016672, and an unrestricted research grant from the Kanzius Research Foundation (SAC, Erie, PA).

## Figures and Tables

**Figure 5:**
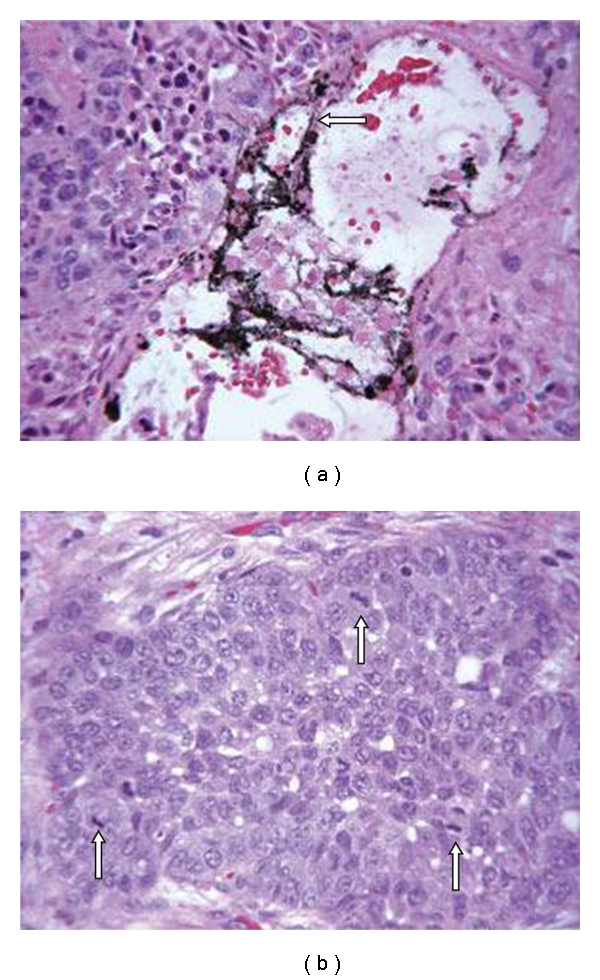
Photomicrographs of hepatic VX2 tumors from rabbits that received (a) or did not receive (b) intratumoral injection of Kentera single-walled carbon nanotubes (SWNTs) followed by 2 minutes of RF field treatment. SWNTs (arrow) can be identified surrounded by necrotic tumor. (Routine H & E stain, magnification, x400). *Reproduced with permission from Cancer, vol. 110, no. 12, pp. 2654–2665, 2007 *

**Table 1:** Non-invasive RF field treatment of Hep3B cells: cell viability was assessed by Propidium Iodide-Fluorescent Activated Cell Sorting (PI-FACS) 18 hours after RF exposure.

RF exposure (min)	Controls Cell death (%)	Pretreated with AuNP (67 *μ*M) Cell death (%)	*P* value
5	75.0 ± 12.2	99.8 ± 3.1	.4
2	21 ± 14.4	98.5 ± 0.5	.001
1	17.6 ± 8.4	99.0 ± 0.2	.001
